# Humanitarian Needs in Government Controlled Areas of Syria

**DOI:** 10.1371/currents.dis.f510b7b5f473a260a215744b4b85c38b

**Published:** 2018-02-15

**Authors:** Shannon Doocy, Emily Lyles

**Affiliations:** Department of International Health, Johns Hopkins Bloomberg School of Public Health, Baltimore, MD, USA; Department of International Health, Johns Hopkins Bloomberg School of Public Health, Baltimore, MD, USA

## Abstract

**Background.:**

Five years of conflict in Syria have led to 13.5 million people in need of humanitarian assistance and 6.6 million internally displaced people. Humanitarian needs are ever-increasing as an inability to maintain humanitarian corridors and ceasefires continue. In light of the protracted nature of the conflict, immense needs, and dearth of large-scale data, we undertook this assessment to inform humanitarian response.

**Methods.:**

A survey of accessible areas, which were largely urban and government controlled, was undertaken from April - June 2016 to identify unmet needs and assistance priorities. A cluster design with probability sampling was used to attain a final sample of 2,405 households from ten of fourteen governorates; 31 of 65 (47.7%) districts were included that are home to 38.1% of people in need (PiN).

**Results.:**

Overall 45% of households received assistance in the preceding month; receipt of aid was lowest in al-Hasakeh (17%). Shelter was a concern, with 48% of households having shelter need(s); the unmet shelter needs were highest in the West Coast, Rif Damascus and al-Hasakeh.  Food security was a major concern where 64% had unmet food needs and 65% at least one indicator of concern; food insecurity was most severe in Rif Damascus and the West Coast. Water was also a concern with 36% of households reporting inconsistent access and 48% no access to water for several day periods; water needs were highest in Aleppo.

**Discussion.:**

This assessment included accessible populations in predominantly urban and government controlled areas, which are likely to have better access to services and fewer needs than populations in rural locations or areas not controlled by the government.  The humanitarian situation in inaccessible and non-government controlled areas is likely to be considerably worse, thus findings should not be generalized. An expanded humanitarian response is desperately needed for Syrians to better endure the conflict.

## Introduction

With an estimated 13.5 million people in need (PiN) of humanitarian assistance and 6.6 million internally displaced people (IDPs), Syria is the country with the world’s largest IDP population and among the most severe ongoing emergencies.^1^^,^^2^ At the start of 2016, of the 13.5 million PiN, 6.5 million (48%) resided in areas controlled by the Government of Syria, 4.5 million PiN were in hard-to-reach areas, and an estimated 8.7 million people had acute needs across multiple sectors.^2^ Humanitarian needs are ever-increasing while an inability to maintain humanitarian corridors and ceasefires continue to limit assistance to populations in areas where needs are greatest.^3^^,^^4^ Many needs remain unmet, and if assistance is provided, it is often insufficient in terms coverage or quantities distributed; for those with multiple needs, assistance may be received in one sector whilst other needs remain unaddressed.^5^^,^^6^^,^^7^

The 2016 Syria Humanitarian Response Plan (HRP) set out three guiding objectives for the humanitarian response: (i) saving lives and alleviating suffering, (ii) enhancing protection, and (iii) building resilience.^8^ While the operational challenges to implementing a widespread response in an environment with poor security and access limitations are well known, financing is another less discussed barrier; only 33% of the nearly US$ 3.5 million needed to fund the 2016 Syria HRP is pledged.^2^^,^^9^ In light of the protracted nature of the conflict, immense humanitarian needs, and dearth of large-scale data, this assessment was undertaken to characterize unmet needs and inform humanitarian response in government controlled areas of Syria.

## Methods

Sample size calculations were based on objectives of identifying unmet needs and assistance priorities and used the most conservative prevalence rate (50%), 80% power (1-β), and design effect of 1.5. A minimum sample of 1600, which allowed for ±3% precision, was increased to 2400 to provide increased power for regional comparisons. Few consistently reported and reliable population figures are available for Syria. A stratified multi-stage cluster design with probability proportional to size sampling was used, both because of challenges in attaining accurate population data and of the desire for region-specific estimates and comparisons. Accessible areas were divided into seven survey areas with, to the extent possible, PiN of similar size.

A 120 cluster x 20 household design was used; clusters were allocated using a stratified approach, where areas with larger PiN were assigned 20 clusters and smaller PiN 10 clusters to allow for similar probability of selection across areas. Within each area, clusters were assigned proportionally at district and sub-district levels using recent population data from the UN Office for the Coordination of Humanitarian Affairs which was perceived to be most reliable.^10^ The assessment incorporated ten of fourteen governorates (Deir-ez-Zor, ar-Raqqa, Idleb, and Quneitra were not accessible), however, not all areas of included governorates were accessible (Table 1).^11 ^In total, 31 of 65 (47.7%) districts were included that are home to 38.1% of PiN and a population of 4.1 million.^2^ Accessible areas were predominantly urban city centers (60%) with fewer clusters in peri-urban areas/remote cities (21%) and rural areas (19%). This distribution is reflective of the predominantly urban population (70%), high levels of urban need, and resulting urban-focused humanitarian response.^8^

**Figure:**
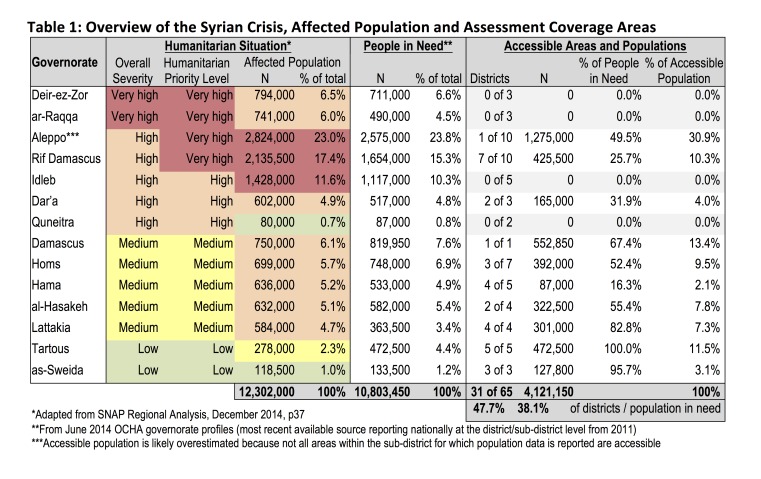
Table 1. Overview of the Syrian Crisis, Affected Population and Assessment Coverage Areas

ARC GIS was used to identify random start points within sub-districts; those in unpopulated areas when reviewing Google Earth imagery were excluded. In developed areas, the nearest intersection, usually within 0.5km, served as the start point; the field team then reviewed start points to ensure accessibility. Every third household in several directions was sampled; replacement sampling was used and no more than two households within an apartment building were included. Back up coordinates were provided and an alternate start point used in the event that planned location was insecure.

To the extent possible, existing content from instruments used with Syrian populations was adapted to improve validity and comparability.^12^^,^^13^^,^^14^^,^^15^^,^^16^ Pilot testing was conducted with Syrian refugees in Lebanon and in Damascus to ensure appropriateness of content and translation. A Training-of-Trainers approach was used where team leaders and study coordinators received five days of training in Lebanon; they later oversaw two days of interviewer training in their respective survey areas. Most interviewers and all team leaders had prior experience conducting humanitarian assessments in Syria.

The assessment was conducted between April and June 2016 by a US-Based international non-governmental organization (iNGO) and a Syrian partner with training and remote support from Johns Hopkins School of Public Health (JHSPH). Interviews ranged from 20 to 45 minutes. To protect anonymity, unique identifiers were not collected and verbal informed consent was used. Data was collected on tablets using the Magpi mobile data platform (Datadyne LLC, Washington, DC). Partner organizations’ staff supervised interviewers and JHSPH performed real-time data review to ensure quality.

Data was analyzed using Stata 13 (College Station, TX) with the ‘svy’ command to account for clustering. Exploratory analysis was conducted to assess if differing non-response rates (0-21%) needed to be accounted for and it was found unnecessary. Summary statistics were not weighted because sampling survey area probabilities were similar and confidence in data used to estimate probabilities low. Sectoral severity scales were developed based on key indicators; cut points were determined by reviewing point estimates and categorizing to attain a distribution. Severity levels were assigned based on select sectoral indicators and the proportion of the population identified as at risk/affected by one or more indicators.

The primary purpose of the assessment was to inform partners’ humanitarian programming and the assessment was conducted by partner organizations’ staff. Permissions to conduct the survey were attained from local community leaders as needed in Syria by partner organizations and survey supervisors. The Johns Hopkins School of Public Health Institutional Review Board determined that JHSPH was not engaged in human research because JHSPH had no interaction with human subjects and was not obtaining identifiable data.

## Results

A total of 2,681 households were approached to participate, of which 10.3% (n=276) declined, yielding a final sample of 2,405 households (response rate=89.7%). The average household head was 51 years old (range 16-103) and 17.7% (CI:15.7-19.8) of households were female-headed. Educational attainment was low with 60.0% (CI:55.9-63.9) of household heads not completing secondary schooling. Less than half (42.7%, CI:37.8-47.7) of households were displaced and 2.0% (CI:1.1-3.5) were returnees. Average household size was 5.1 (CI:4.9-5.3, range 1-22). A majority of households (65.4%, CI:61.9-68.7) had children ≤17 years and 29.3% (CI:26.3-32.4) had children <5 years of age; 37.1% (CI:34.3-40.0) had older adults. The population age distribution is presented in Figure 1. The most common vulnerable group was those with chronic health conditions, reported by 43.3% (CI:40.5-46.1) of households; 12.6% (CI:11.1-14.2) had disabled members and 7.7% (CI:6.5-9.2) had pregnant or lactating women.

**Figure:**
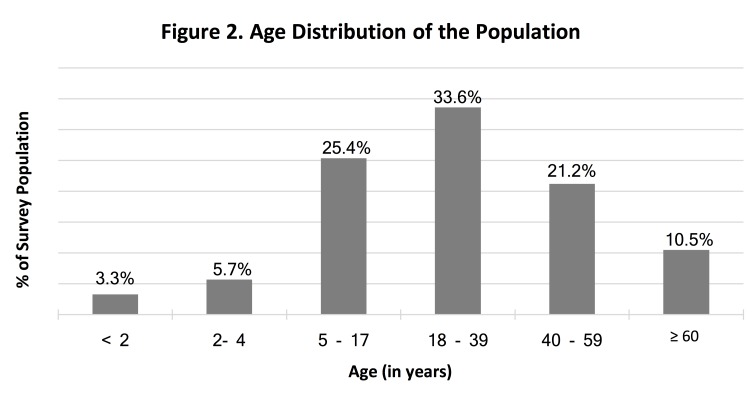
Figure 2. Age Distribution of the Population

**Humanitarian Assistance and Unmet Needs.** Humanitarian assistance was received by 45.1% of households in the preceding month; only 11.6% received multiple types of aid (Table 2, Figure 2). The most frequent assistance received were food items (42.7%) and hygiene kits (6.4%); ≤2.5% received aid in all other categories. Receipt of assistance differed significantly by region: more than half (52-57%) of households in Aleppo, Rif Damascus, the South, and the Central areas received assistance compared to 35% in Damascus and West Coast and 16.5% in al-Hasakeh (p<0.001). Unmet needs were nearly ubiquitous with 96.5% reporting one or more unmet need. The most frequently reported priority needs included more food (29.4%), rent support/improved shelter (15.4%), health services/medications (11.2%), improved security (10.8%) and better quality food (9.4%). Food (64.1%), non-food items (NFIs) (29.3%), health (26.8%), and shelter (24.4%) were the sectors with highest reported unmet needs. The proportion of households with unmet needs was similar across regions (p=0.208); however, priority needs varied by location (p<0.001). Of note were the large proportions in al-Hasakeh and Aleppo reporting food (70.0% vs 38.8% overall) and security (43.5% vs. 10.8% overall), respectively, as priority needs.

**Figure:**
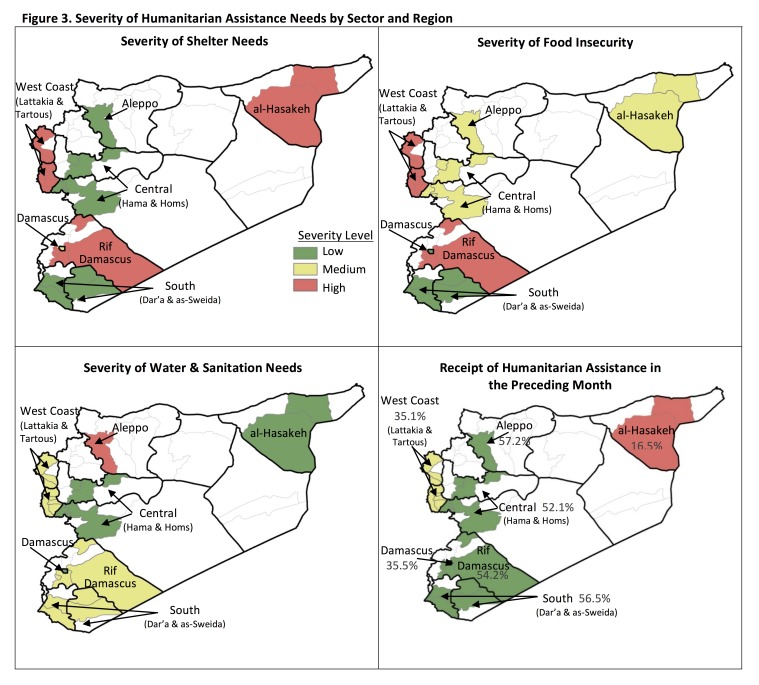
Figure 3. Severity of Humanitarian Assistance Needs by Sector and Region

**Figure:**
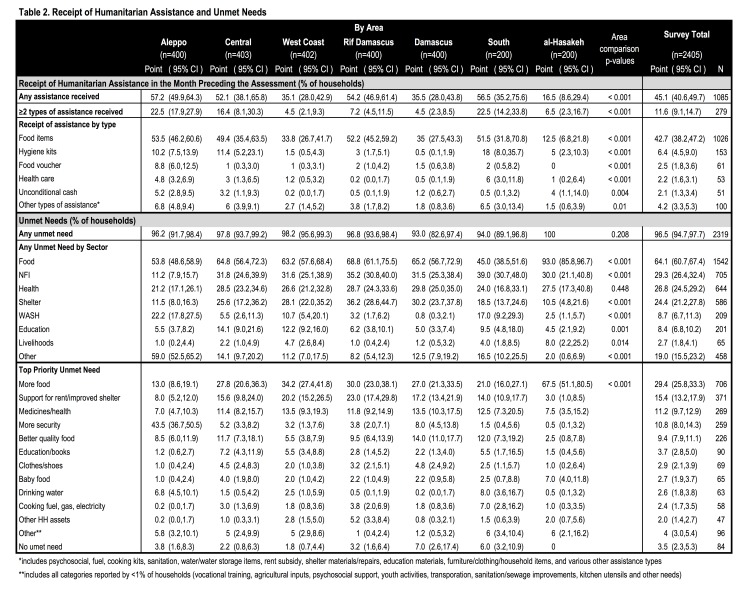
Table 2. Receipt of Humanitarian Assistance and Unmet Needs

**Shelter.** Most households resided in an unshared houses or apartments (89.2%) with smaller proportions residing in unfinished buildings/construction sites/warehouses (4.8%), rented rooms (3.0%), or other accommodations (3.0%) (Table 3, Figure 2). More than half owned (56.3%) and many rented (31.8%) or were hosted (10.2%). Over half (62.1%) of households reported dwellings in good condition and 37.9% had a concern about their dwelling or needed shelter repairs; the most frequent problems included high humidity (27.0%), water leakage (10.4%), and poor ventilation (7.3%). Crowding was not a major concern; only 11.6% of households reported ≥5 people per sleeping room. Differences by geographic area were significant. The greatest need for repairs was in the West Coast (63.2%), crowding was most frequent in al-Hasakeh (30.5%), and a high proportion of Rif Damascus households (13.8%) lived in construction sites, unfinished buildings, or warehouses. Overall, shelter needs were greatest in the West Coast, al-Hasakeh, and Rif Damascus where more than half of households had at least one shelter indicator of concern.

**Figure:**
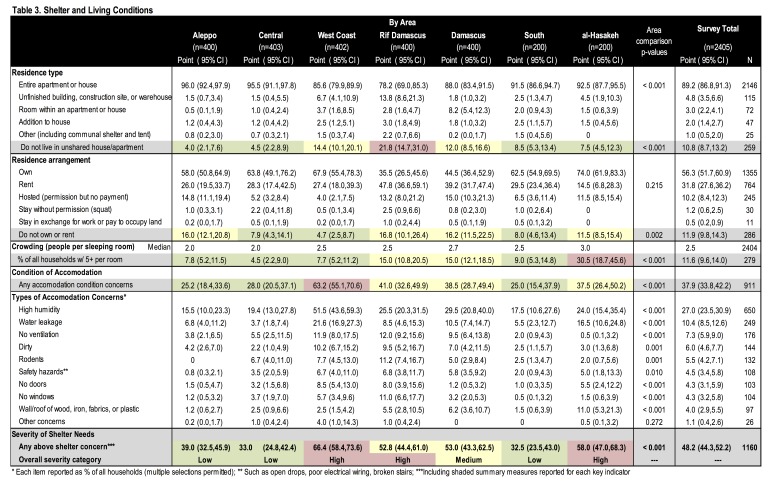
Table 3. Shelter and Living Conditions

**Food Security.** Food security was assessed using the Food Consumption Score (FCS);^12^ 7 ^11^5.9% of households had an acceptable FCS (mean=55.2) (Table 4, Figure 2). Lack of food or insufficient means to buy food in the preceding month was reported by 38.1% and 56.7% reported no food stocks. Food assistance reliance was low with 72.4% reporting <25% of their diet from aid. Use of any and extreme coping strategies[1] in the preceding month were reported by 84.9% and 54.3% of households, respectively. Most frequent were reliance on less preferred/expensive foods (54.0%), spending savings (53.5%), credit/borrowing (40.1%) and reduced portion size (34.4%). A minority (12.8%) spent >75% of total expenditures on food. There were statistically significant differences in food security by region. Using FCS, food insecurity was highest in Rif Damascus, the Central and West Coast survey areas where 31.1-34.8%of households did not have an acceptable FCS. Both Rif Damascus and the West Coast had high prevalence of coping mechanism use with 67.5%-71.9% using extreme coping strategies. In al-Hasakeh, nearly half (47.7%) reported that >75% of total expenditures on food. Overall, food insecurity was most severe in Rif Damascus and the West Coast, moderate in Aleppo and the Central area, and lowest in Damascus and the South. In all areas, half to three-quarters (49.0-78.4%) of households were food insecure by one or more indicator making food insecurity the area of greatest unmet need.

**Figure:**
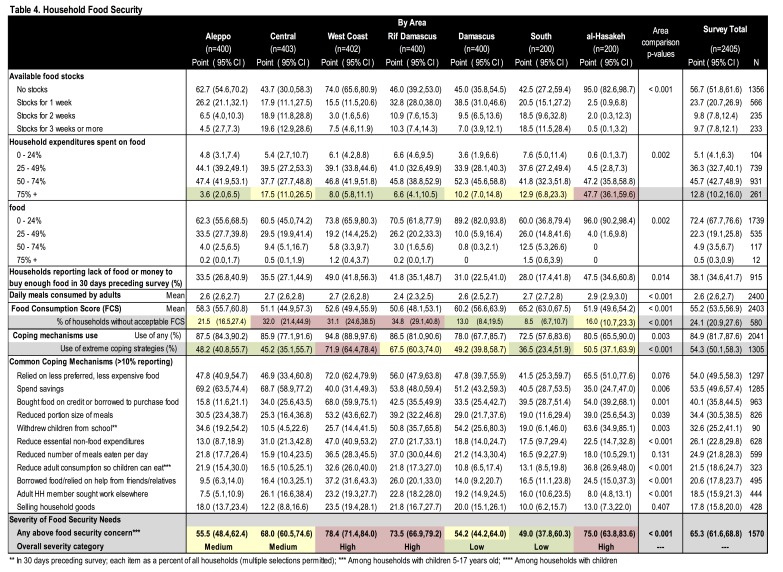
Table 4. Household Food Security

**Water & Sanitation.** The most frequent drinking water source was an inside tap supplied by municipal water networks (79.6%) followed by paid tanker/truck water (11.8%); similarly, an inside tap was also the most frequent source of water for other purposes (90.4%) (Table 5, Figure 2). Water access was fair with 36.4% reporting not having access to water 24 hours a day; those without regular access reported having water for 12 hours daily. When running water was not available, sources included stored water (49.3%) or trucked water (34.3%); nearly all (97.9%) were able to store water and 72.5% had storage capacity >500L. Water access was perceived as a problem by 72.2% of households and nearly half (48.0%) reported no access for several days at a time in the three preceding months. With respect to sanitation, flush toilets (45.6%) and improved latrines (40.3%) were the most common; 18.1% of households did not have improved sanitation and 5.9% reported sharing toilet facilities.^17^ Water and sanitation indicators differed significantly by region. The need for improved water access and water storage was greatest in Aleppo where the majority (88.8%) did not have continuous access to running water and 95.5% experienced several days without water in the 3 preceding months. Sanitation was the worst in the West Coast and Rif Damascus where 27.1% and 21.7% of households, respectively, lacked improved sanitation.

**Figure:**
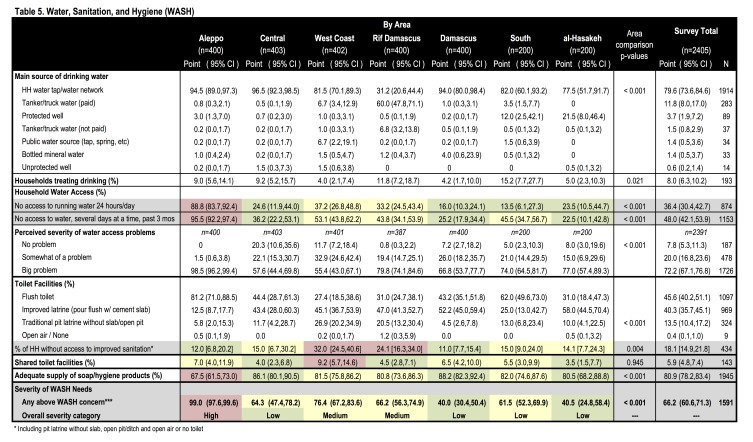
Table 5. Water, Sanitation, and Hygiene (WASH)

[1][1] Including reducing portion size; reducing number of meals eaten per day; reduced adult consumption to allow children to eat; restricting consumption of female household members; going entire days without eating; selling household assets, productive assets, house or land; withdrawing children from school; involving children in income generation; engaging in high risk/socially degrading jobs; sending members to eat elsewhere; and child marriage.

## Discussion

To our knowledge this is the only recent quantitative multi-sectoral assessment that covers a significant proportion of Syria. The assessment was undertaken in areas that were predominantly government controlled which are less likely to have experienced direct effects of conflict, such as violence and infrastructure destruction, than inaccessible and non-government controlled areas. Basic services, such as health, education and utilities are likely to be more accessible and functioning in in areas included in the assessment as compared to elsewhere in Syria; furthermore, the participants were from predominantly urban areas which are likely to have better access to services than rural areas. Assessment findings should not be generalized to Syria more broadly because of contextual differences and it is likely that humanitarian needs in non-government controlled areas are significantly greater than in the locations included in this assessment.

The ability to compare results with other sources is limited because accessible areas and measurement methods vary widely. The 2016 Humanitarian Needs Overview (HNO) and Syria Dynamic Monitoring Reports (DMR) have similarly wide geographic coverage and report on all sectors, but rely on secondary data or assorted other methodologies.^18^^,^^19^ A number of sector-specific assessments and program evaluations are available, but there were no peer reviewed publications with primary data collected in 2015 or 2016.^20^^,^^21^^,^^22^^,^^23^

Shelter needs were greatest in the West Coast, Rif Damascus, and al-Hasakeh, though concerns differed greatly by region. Residing in construction sites or warehouses was common in Rif Damascus whereas dwelling repairs and crowding were predominant concerns in the West Coast and al-Hasakeh, respectively. One potential reason for the high levels of crowding in al-Hasakeh is that displacement in al-Hasakeh was more recent than in other survey areas, where 69% of IDPs were displaced in/after 2015 compared to <10% in other survey areas. The June 2016 DMR indicated shelter needs were most severe in Rif Damascus, aligning with findings from this assessment; Homs also had higher shelter needs in the DMR. Comparison with other recent assessments suggest that our findings may underestimate shelter needs; in this assessment 38% were found to lack adequate shelter compared with 79% in the DMR and 59% reported by OCHA.^19^^,^^24^ ^18^

Food security was a major concern. Despite high levels of assistance, 64% of households had unmet food needs which is greater than our 2014 survey where 50% reported unmet food needs.^23^ Food insecurity was greatest in Rif Damascus and the West Coast; Aleppo and the Central area were moderately food insecure and Damascus and the South were areas of lesser concern. Findings are relatively aligned with other sources; 85% of households reported using negative coping strategies compared to 79% in a June 2016 food security review.^25^ Food consumption, which was acceptable in 76% of households, was slightly better than in a 2015 WFP report where only 65% had an acceptable FCS; this is difference is not likely to reflect a situational improvement and is probably results from differences in coverage areas.^22^^,^^23^ High food prices and receipt of insufficient quantities of food, both previously identified concerns, are likely contributing factors to the observed high food expenditures and food insecurity.^18^^,^^22^^,^^26^^,^^27^

^17,21,25^More than one-third (36%) of households did not have consistent access to running water and 48% reported no access to water for several day periods. Water access was worst in Aleppo and reported to be a problem by 72% of households overall. Sanitation was a lesser concern with 86% having improved sanitation and little sharing (6%); sanitation needs were greatest in the West Coast and Rif Damascus. Findings from this assessment, where Aleppo had the greatest WASH needs, are supported by previous assessments; however, Hama and Homs were ranked as having similarly high WASH needs in the HNO but moderate needs in this assessment.^10^^,^^18^ Of note, our assessment reports on access to water but could not assess water quality which is also a known concern, where 70% of Syrians are estimated not to have access to safe drinking water.^18^

**Limitations.** Triangulation and the stratified design may have reduced sampling bias, but given the limitations of available population data and ongoing displacement, it is likely the sample is unrepresentative. Many areas were inaccessible, thus findings are not nationally representative and probably present a better-than-actual characterization of the situation where the most severely affected areas with the greatest unmet needs were inaccessible; this was especially true in Aleppo, one of the most severely impacted areas, where the majority of the city was inaccessible.^28^ The training-of-trainer method, particularly given the extended period between trainings, and use of paper questionnaires in some locations may have contributed to poor data quality. Because of length limitations; key sectors including health, NFIs, education and protection were not assessed in sufficient depth and had limited indicators that could be used to develop severity scales and thus are not presented.

## Conclusions

Timely and accurate report of needs in emergencies is a persistent challenge. Situational reporting is often anecdotal or based on information provided by convenience samples, key informants, projections or a combination of these approaches. This assessment is the only large-scale multi-sectoral assessment conducted in Syria in the past two years that uses a random sample of households, thus findings are more scientifically rigorous than most other available sources.

The greatest levels of unmet humanitarian needs were observed in the West Coast, Rif Damascus, and al-Hasakeh. Of note, was the finding that Lattakia and Tartous had high unmet needs in both the shelter and food security sectors and moderate water and sanitation needs which contrasts with other recent reports indicating lower levels of need in those governorates.^18^ Lattakia and Tartous are mostly government controlled, however, the proportion of households receiving assistance was among the lowest of all survey areas which may explain the higher than anticipated levels of unmet need. Al-Hasakeh differed from other areas included in the assessment in that displacement was more recent and coverage of humanitarian assistance was low, which is largely due to aid organizations having poor access (though indications are this may be improving). The high prevalence of coping mechanism use and other indicators suggest the population in al-Hasakeh is in need of additional support and that an expanded response could prevent future deterioration.

When considering these findings, it is important to note they are representative of accessible populations in predominantly urban areas which are likely to have better access to services and fewer needs than populations in rural locations or areas not controlled by the government. The humanitarian situation in inaccessible areas is likely to be considerably worse, thus findings presented here likely underestimate the true scope of humanitarian needs which will continue to exceed response capacity, both due to access limitations and funding shortfalls.^18^ Extreme destruction and violence, widespread humanitarian law violations, and lack of progress towards peace portend a deteriorating situation. Sustained support from the international community and an expanded humanitarian response are desperately needed for Syrians to better endure the conflict.

## Competing Interests

Shannon Doocy is on the Editorial Board at PLoS Currents. There are no other conflicts of interest.

## Funding Disclosure

The study was funded by international donor contributions for humanitarian operations by a US-Based international non-governmental organization that wishes to remain unnamed. The funders had no role in study design, data collection and analysis, decision to publish, or preparation of the manuscript.

## Corresponding Author

Shannon Doocy: doocy1@jhu.edu

## **Data Availability**

Minimal underlying data for this manuscript is deposited publicly in the Humanitarian Data Exchange and can be accessed at: https://data.humdata.org/dataset/humanitarian-needs-in-government-controlled-areas-of-syria
